# The effect of oral chronic graft-versus-host disease on bodyweight: A cohort study

**DOI:** 10.1371/journal.pone.0293873

**Published:** 2024-01-18

**Authors:** Ali Anwar Aboalela, Fathima Fazrina Farook, Norah N. Alazaz, Nada Alshahrani, Aalia Alharthi, Roa Hagr

**Affiliations:** 1 Maxillofacial Surgery and Diagnostic Sciences Department, College of Dentistry, King Saud bin Abdulaziz University for Health Sciences, Riyadh, Saudi Arabia; 2 Ministry of National Guard-Health Affairs, Riyadh, Saudi Arabia; 3 King Abdullah International Medical Research Center, Riyadh, Saudi Arabia; 4 Preventive Dental Science Department, College of Dentistry, King Saud Bin Abdulaziz University for Health Sciences, Riyadh, Saudi Arabia; 5 College of Dentistry, King Saud bin Abdulaziz University for Health Sciences, Riyadh, Saudi Arabia; 6 College of Dentistry, Princess Nourah Bint Abdul Rahman University, Riyadh, Saudi Arabia; University of Missouri, UNITED STATES

## Abstract

**Aim:**

This retrospective cohort study aimed to evaluate the association between body weight and oral cGVHD (chronic graft versus host disease).

**Methods:**

Patients with oral cGVHD were compared with an age and gender-matched non-GVHD cohort in terms of demographic information, body mass index (BMI), date of transplant, length of hospitalization, and oral complications. Weight was stratified in pre-and post-transplant weight, mean weight after acquiring cGVHD for the first year, and post-oral cGVHD BMI. Each patient was matched and compared with two controls at a 1:2 ratio. Firth’s penalized likelihood logistic regression was used to investigate the association between oral complications and weight loss greater than 5% in the oral cGVHD group.

**Results:**

This study included 137 patients (n = 42 oral cGVHD, n = 12 non oral-cGVHD and n = 83 non-GVHD). The oral cGVHD cohort had a 1.44 times higher risk (RR) of being underweight (BMI<18.5 kg/m2) compared to the non-GVHD cohort. Oral mucositis was an independent predictor of weight loss above 5% in the oral cGVHD cohort (p < 0.001)

**Conclusion:**

The weight loss was more prevalent among oral cGVHD, and oral mucositis was linked to significant weight loss. Weight loss may indicate the need to initiate early and aggressive symptomatic oral cGVHD treatment.

## Introduction

Hematopoietic stem cell transplantation (HSCT) is a well-known procedure to treat both malignancies and non-malignant disorders, including hematological, other metabolic, and immunodeficiency disorders [1]. Although an increasing number of patients receive this successful treatment, graft-versus-host disease (GVHD) remains the main complication with unsatisfactory results [1–3]. Acute GVHD (aGVHD) is characterized by affecting the skin, liver, and gastrointestinal tract [4]. The chronic form, chronic graft-versus-host disease (cGVHD), is a serious complication of allogeneic hematopoietic cell transplantation (HCT), that occurs in 30% to 70% of the patients [5]. It can affect any organ in the body but mostly affects the skin, mouth, gastrointestinal tract, liver, lungs, musculoskeletal system, and eyes [6]. Chronic GVHD may decrease quality of life, impair function, and lead to the ongoing need for immunosuppressive medications and associated complications [6].

Although cGVHD can affect multiple organs, oral cGVHD is the second most frequently involved organ system [7] with a prevalence of 45% to 83%. It occurs more frequently in adult patients than in children [7–10]. cGVHD can manifest itself in a variety of ways, depending on the type and severity of tissue alterations, and it can affect any part of the oral cavity [8]. Oral cGVHD can present as mucosal lesions, salivary gland dysfunction, and musculoskeletal involvement [7, 11]. Oral mucosal lesions are characterized by lichenoid features including erythema, ulcerations, and hyperkeratosis [12]. Any site in the oral cavity can be affected including the gingiva usually presenting as desquamative gingivitis. The most usually implicated sites are the buccal and labial mucosa, as well as the lateral borders of the tongue [13]. Affected sites can range from symptoms of mild sensitivity to severe pain, depending on the type of involvement, preventing normal daily function. Additionally, certain types of food can be irritating leading to both avoidance and a lack of interest in eating. Salivary gland dysfunction can present clinically as mucoceles or xerostomia. Complications include dysphagia, opportunistic infections, and, altered taste sensation due to the altered quality and quantity of saliva. Musculoskeletal cGVHD of the oral cavity can also limit mouth opening and have a further impact on dietary function and hygiene. Overall the combined effects of mucosal, salivary, and musculoskeletal cGVHD can be summarized into clinically significant oral symptoms, such as oral pain, oral sensitivity, reduced mouth opening, dry mouth, and taste changes to normally tolerated foods and drinks [14]. These symptoms can result in both a significantly decreased oral intake and a nutritionally deficient caloric diet. The effect on the overall health and survival of patients is evident and can lead to an increased burden on the health care system [8–10, 15, 16].

Evidence shows that patients with cGVHD have a significantly lower BMI associated with poor nutritional status [17, 18] and weight loss. Malnutrition is a clinically significant problem in this population that contributes to functional and health status impairment in transplant survivors [18]. The effect of oral cGVHD and the related symptoms on these clinically challenging problems must be investigated. Studies reported that the clinical manifestations associated with weight loss in this population include erosions, ulcerations, xerostomia, decreased salivary flow, oral infections, oral and esophageal pain, diarrhea, dysphagia, vomiting, sensitivity, and medication used in the treatment of HSCT [9, 11, 15, 17–20].

The limited knowledge with regards to the relationship between body weight and oral cGVHD, and the need for a better understanding of malnutrition caused by oral manifestations in patients suffering from cGVHD, both hinder the ability to develop more efficient preventative and therapeutic strategies and further underpin the need for the current study. This study aimed to measure the association between body weight and oral cGVHD in post-allogeneic HSCT patients, as well as the impact of oral complications on malnutrition (underweight).

## Methods

The study protocol was approved by the Institutional Review Board at King Abdullah International Medical Research Centre (KAIMRC) (SP20/478/R). All the clinical charts from patients who received an allogeneic myeloablative HSCT at King Abdulaziz Medical City (KAMC), a tertiary care hospital in Riyadh, Saudi Arabia, from January 2015 to December 2020, were retrieved from the electronic medical record database Best Care system. Two cohorts of patients, aged 16 to 70 years, were defined as GVHD and non-GVHD. The GVHD cohort was further subdivided into two subcohorts defined as oral cGVHD and non-oral-cGVHD. Eligibility for the oral cGVHD cohort required a confirmed diagnosis of oral cGVHD based on the 2014 Diagnosis and Staging Working Group Report and the non-oral-cGVHD a confirmed absence of oral cGVHD [6]. Eligibility for the non-GVHD cohort were patients receiving an allogeneic HSCT transplantation who did not develop acute or chronic GVHD. All the patients had a weekly clinical examination during the first 3 months, followed by clinical evaluations every 2 weeks during the first year and monthly thereafter.

**Fig 1:**
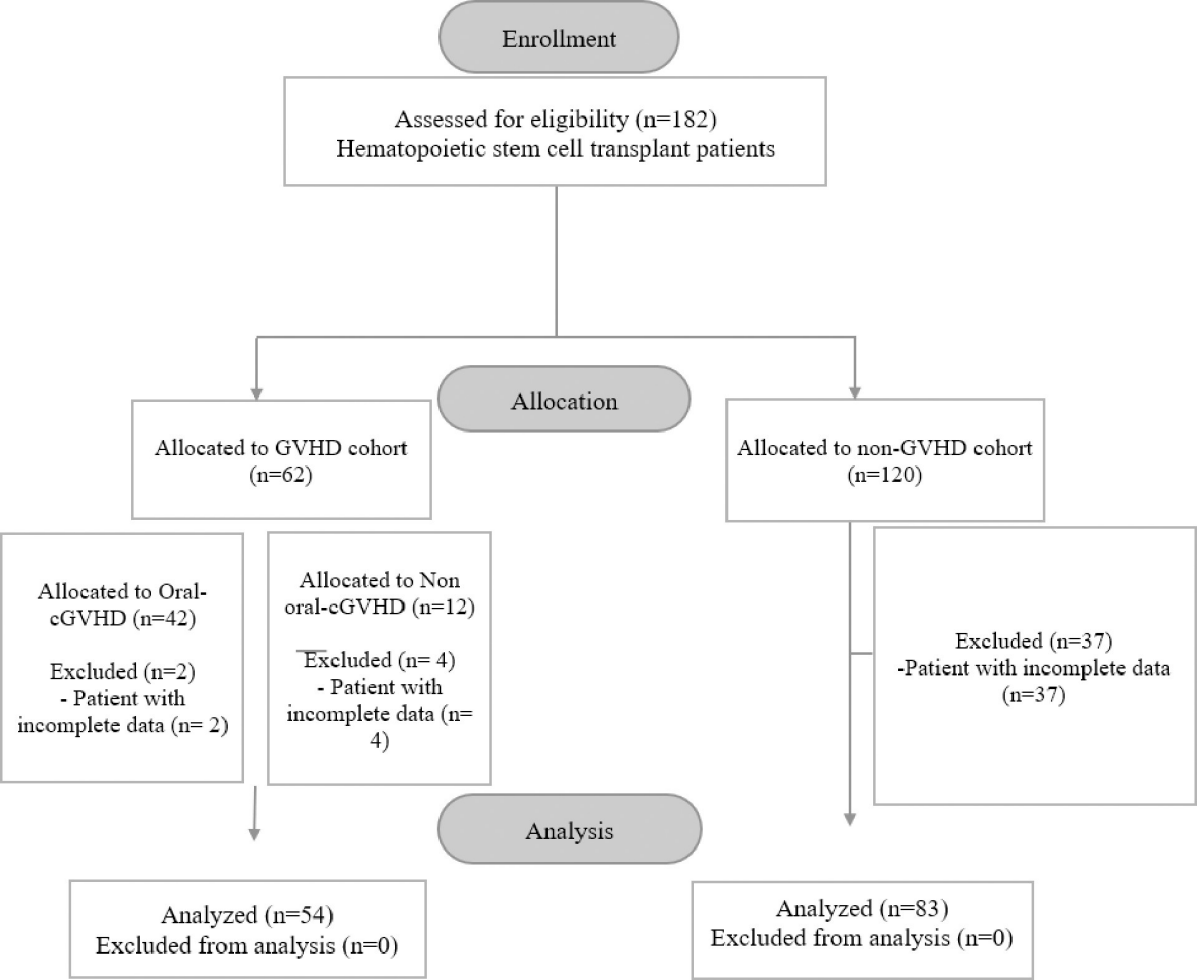
Flow chart of study design.

All the patients with confirmed oral cGVHD were included at a 1:2 ratio to age and gender-matched non-GVHD cohort. We based our required sample size calculation on pilot data that indicated a proportion of 40% malnourishment in oral cGVHD, and 15% malnourishment in the non-GVHD cohort. The OpenEpi sample size calculator for cohort studies was used to calculate the sample size [21]. Assuming that the percentage of exposed with the outcome is 40% and to detect an odds ratio of 3.8 with a power of 80%, 95% confidence interval, and a significance level of 5%, a minimum of 41 cases with oral cGVHD and 82 non-GVHD cohorts were required. 182 hematopoietic stem cell transplant patients were considered eligible for the study. However, the final sample for this study comprised 54 individuals from the GVHD cohort and 83 individuals from the non-GVHD cohort. 43 patients were excluded from the analysis as their weight had not been recorded at certain time points (Fig 1).

The study was retrospective and all data were fully anonymized.

All the variables related to oral cGVHD were validated by trained personnel. The inclusion criteria included patients aged ≥16 years and a diagnosis of oral cGVHD by the primary physician in the electronic database system. Pediatric patients and patients with incomplete or missing data were excluded. Due to the heterogeneity of the charts, the data abstraction fields were restricted to demographic variables, baseline disease, transplantation information, cGVHD characteristics, as well as weight records, and oral complications. The BMI and weight were stratified and collected as pre-transplant and post-transplant weight, mean weight after acquiring cGVHD for the first year, and post-cGVHD BMI.

The subset of the oral cGVHD cohort was also identified by the presence or absence of oral complications occurring during transplant and were recorded up to 1 year post transplant. Oral complications that were observed and recorded in the oral cGVHD subset included oral infections such as oral candidiasis, oral pain, and dry mouth (xerostomia). In addition, dysphagia was recorded when the patient experienced difficulty during the act of swallowing, while odynophagia was recorded when the patient experienced pain during the act of swallowing. Dysphagia and odynophagia may have occurred concurrently or separately during the course of follow up. Other oral lesions, such as oral mucositis and stomatitis were documented. Oral mucositis was recorded following a fairly predictable clinical and chronological course of mucosal injury (erythema and/or ulcerations) in response to HSCT conditioning regimens, and generally resolving within 3 weeks. Oral ulcerations becoming worse with engraftment and white blood cell count recovery were further investigated for the possible emergence of aGVHD. Stomatitis was recorded when ulcerations developed, secondary to mTOR inhibitors [22]. They mostly appeared ovoid with white/grey central areas, and generally resolved within 10–14 days of development.

The non-oral chronic GVHD manifestations were recorded and confirmed in a hospital setting by a multidisciplinary team that included hematologists, oncologists, and clinicians from different related specialties according to site of involvement. Information was then documented according to NIH guidelines.

The demographic information and baseline data of the non-GVHD cohort were also documented in terms of age, gender, BMI, date of transplant, primary disease, and length of hospitalization. The BMI and weight were classified the same as for the oral cGVHD cohort.

The percent weight change was calculated as the difference between the weight at the clinic visit and the weight pre-SCT, divided by the weight pre-SCT. Patients were dichotomized into two different groups: (a) weight gain or 5% weight loss, and (b) >5% weight loss.

### Statistical analysis

All analyses were performed using NCSS software version 2020. Descriptive statistics were generated for all the baseline data. The continuous variables are described as mean and standard deviation (SD) and frequency and percentage for the categorical variables. The prevalence per 100 patients and IRs per 100 person-years with a 95% confidence interval (CIs) and the risk ratio were calculated. In the analysis, quasi-complete separation led to an infinite maximum likelihood (ML) estimate of the effects of the oral symptoms. Firth’s penalized likelihood logistic regression was used to determine the association between oral complications and weight loss above 5% in the oral cGVHD cohort, to address potential issues due to the small sample size, and quasi-complete separation. Statistical significance was based on a p-value of 0.05 or less.

## Results

This study included 137 patients (n = 42 oral cGVHD, n = 12 non oral-cGVHD and n = 83 non-GVHD). The median age was 30 years, with a range of 16 to 69 years. Gender was equivalent (p = 0.09). The median time since SCT was 3.5 years. The most frequent indication for the transplant was Sickle Cell Anemia (SCA). The characteristics of the sample are listed in detail in Table 1 and the characteristics of the matched cohorts in Table 2.

**Table 1:** Patient and cGVHD characteristics.

Demographic variables	
**Total no. of patients**	137
**Gender**	78 M/ 59 F
**Age at visit (years)**	
**Median**	30
**Range**	16–69
**Time since stem cell transplant, years (median)**	3.61(1.74)
**Median**	3.5
**Range**	0.35–10.5
**Length of hospitalization**	5.67(3.85)
**Primary disease**	
Sickle Cell Anemia (SCA)	50 (40.00)
Acute lymphoblastic leukemia (ALL)	28 (22.4)
Acute Myeloid Leukemia (AML)	23(18.4)
Thalassemia and SCA	6 (4.80)
Hodgkin’s lymphoma (HL)	5 (4.00)
AA (Aplastic anemia)	5 (4.00)
Multiple Myeloma (MM)	3 (2.40)
Chronic Myelogenous Leukemia (CML)	2 (1.60)
**T cell Prolymphocytic Leukemia (T-PLL)**	2 (1.60)
Paroxysmal Nocturnal Hemoglobinuria (PNH)	1 (0.80)
**Organ involvement in the oral cGVHD cohort**	n*(%)
**Skin**	31(73.8)
**Liver**	24 (57.14)
**Eyes**	22(52.38)
**GI tract**	5(12)
**Lungs**	5(12)
**Joint/fascia**	4(9.52)
**Genital area**	1(2.63)

*number of at least one given organ involvement in the oral cGVHD cohort

**Table 2:** Characteristics and outcomes of the matched cohorts.

Characteristics	cGVHD (n = 54)	NonGVHD (n = 83)	P-value
Male sex, no (%)	25	41	0.481
Age, y*	27	30	0.706

*Median (data expressed)

BMI was defined as the weight (kg) divided by the square of height (m) and classified as underweight (BMI < 18.5 kg/m^2^) or normal/above normal weight (BMI >18.5 kg/m^2^) [23]. The results highlight that the oral cGVHD underweight group (BMI<18.5) had a higher risk of being underweight than the non-GVHD cohort.

The prevalence (95% CI) per 100 patients of being underweight was 38.1 (23.6–54.4) in the oral cGVHD cohort and 26.5 (17.4–37.3) in the non-GVHD cohort. The IR (95% CI) per 100 patients being underweight was 28.6 (14.8–49.9) in the oral cGVHD cohort and 8.43 (3.4–17.4) in the non-GVHD cohort. The group who had oral cGVHD had 1.44 (RR) times the risk of being underweight compared to the non-GVHD group. For the weight change analysis, the percent weight change was calculated as the difference between weight at the clinic visit and weight pre-SCT, divided by weight pre-SCT. Patients were stratified in two groups: (a) weight gain or <5% weight loss, (b) >5% weight loss. Table 3 displays the incidence of each cohort in each weight change category. The statistically significant factor in the oral cGVHD cohort was that the majority of patients were in the >5% weight loss category. The non-GVHD cohort approached statistical significance, with a higher percentage of patients in the <5% weight loss category.

**Table 3:** Two cohorts stratified by weight category.

	Weight gain/<5% loss n (%)	>5% weight loss n (%)	Total n (%)
Oral cGVHD cohort	12(28.57)	30(71.43)	42(100)
Non-GVHD cohort	64(77.11)	19(22.89)	83(100
Total	76	49	125

Logistic regression was performed to ascertain the effect of oral involvement in cGVHD patients on the likelihood that participants have >5% weight loss. The logistic regression model was statistically significant, χ^2^(1) = 4.14, *p* < .05. The model explained 7.0% (Nagelkerke *R*^*2*^) of the variance in body weight and correctly classified 70.4% of cases. Oral cGVHD subjects were 3.94 times more likely to exhibit >5% weight loss than non-oral cGVHD subjects.

A chi-square test of independence to examine the relation between mucositis and > 5% weight loss showed that there is a significant relationship between the two variables. Subjects with oral mucositis were more likely than non-oral mucositis subjects to have >5%weight loss, *X*^2^ (1, *N* = 42) = 18.04, *p* < .001. Firth’s Logistic Regression Analysis was performed by including the oral symptoms, oral mucositis was found to be statistically significant (p < 0.001). Oral mucositis was an independent predictor of weight loss above 5% in the oral cGVHD cohort. The odds ratio for oral mucositis was 23.2 (4.8–59.3), p < 0.001. The other oral features were not significant predictors of weight loss in oral GVHD (Table 4).

**Table 4:** Unadjusted odds ratios (uOR), adjusted odds ratios (aOR), and 95% confidence interval (95% CI) of the oral complications associated with >5% weight loss in patients with oral graft-versus-host disease.

	Unadjusted odds ratio			Adjusted odds ratio		
Variable	uOR	95% CI	P-value	aOR	95%CI	P-value
**Oral infection**	1.86	0.42–11.05	0.457	1.03	0.13–8.26	0.981
**Stomatitis**	0.30	0.07–1.26	0.111	1.02	0.16–7.99	0.982
**Dysphagia**	0.55	0.10–2.97	0.134	1.14	0.17–8.88	0.140
**Mucositis**	23.22	4.77–159.31	0.0005	17.17	3.36–127.39	0.0003
**Oral pain**	8.32	0.90–173	0.182		0.07–163.25	0.918
**Xerostomia**	1.78	0.31–18.65	0.576	1.48	0.12–27.65	0.772
**Odynophagia**	0.32	0.04–2.35	0.297	0.39	0.03–5.59	0.191

When Firth’s Logistic Regression Analysis was performed to check the contribution of involvement with other organs/systems to the bodyweight, by including the involvement of oral cGVHD with and without the GI system (GI tract and/or liver) involvement, neither of the involvements were found to be different significantly (p > 0.001) (Table 5).

**Table 5:** Unadjusted odds ratios (uOR), adjusted odds ratios (aOR), and their respective 95% confidence intervals (95% CI) of the involvement of oral cGVHD with and without GIT associated with >5% weight loss in patients with oral graft-versus-host disease.

	Unadjusted odds ratio			Adjusted odds ratio		
Variable	uOR	95% CI	P-value	aOR	95%CI	P-value
**Oral and GIT and liver**	0.82	0.19–3.16	0.78	0.49	0.003–7.082	0.639
**Oral and other**	0.93	0.24–3.93	0.93	0.51	0.003–8.148	0.666

## Discussion

This is the first study to show that oral symptoms can be considered as potential factors related to weight loss in patients with oral cGVHD was a statistically significant factor (p < 0.001). Acute oral mucositis a distinct condition occurring as the result of chemoradiotherapy conditioning regimens for allogeneic HSCT and resolve completely before the development of cGVHD, This specific finding was found to be an independent predictor of weight loss above 5%. Acute oral mucositis can first present as erythema shortly after infusion and progress to painful ulcerations with pseudomembranous formations preventing oral intake, and resulting in both malnutrition and weight loss [24, 25]. Acute oral mucositis generally resolves 3 weeks post-infusion. The most likely cause of not associating both conditions in previous studies is the timegap between resolution of acute oral mucositis and cGHVD.

Another important finding was that the underweight (BMI<18.5) group had a higher prevalence rate in the oral cGVHD cohort than the non-GVHD cohort. The oral cGVHD had 1.44 times the risk of becoming underweight compared to the non-GVHD cohort. The number of patients in the >5% weight loss category in the oral cGVHD cohort was statistically significant. Moreover, the oral cGVHD subjects were 3.94 times more likely to exhibit >5% weight loss than non-oral cGVHD subjects. The possibility of becoming underweight in the oral cGVHD cohort could be explained by the presence of erosions and ulcerations in the oral cavity as a manifestation of cGVHD, oral infections, and medications prescribed for HSCT and in the management of cGHVD. All factors may contribute to decreased oral intake and weight loss [26]. Similarly, a study done by Fassil et al. found that oral pain, sensitivity, and patient self-identified symptoms contributed to a decline in calorie intake [19]. In the current study, 71% of the oral cGVHD patients had more than 5% weight loss (Table 3). This result is similar to a study by Jacobsohn et al. [18], reporting that 75% of cGVHD patients will have 5–10% weight loss. Our study shows that 38% of the oral cGVHD patients were malnourished, which is relatively higher than a study conducted by Bassim et al., which reported that 29% were malnourished in patients with cGVHD [17].

In this study, SCA was the most common indication for transplantation. Although there is limited information available about SCA prevalence in Saudi Arabia, studies report that SCA is a relatively common genetic disorder here. Depending on the area, carrier status ranged from 2% to 27%, and as high as 1.4% had SCD. The Eastern province has the highest prevalence, followed by the southwestern provinces [27–29].

We also examined the relationship between weight loss in oral cGVHD patients and oral complications that could be contributing to weight loss. Interestingly, oral complications such as oral infections, stomatitis, dysphagia, oral pain, xerostomia, and odynophagia do not appear to be related to weight loss. Our findings agree with a study done by Jacobsohn et al. reporting that symptoms such as oral pain, oral sensitivity, and odynophagia did not appear to be related to weight loss in cGVHD patients [18]. In a study by Imanguli et al. [11], after adjusting for cGVHD severity, patients with salivary involvement also had a significantly lower BMI, compared with patients with an intact salivary flow rate, contrary to our findings that xerostomia did not have any contribution to weight loss in oral cGVHD patients.

Mucositis, one of the main side effects that occur after conditioning regimens, is an inflammatory-driven process of the oral mucosa and was a statistically significant factor in oral cGVHD patients in our study. Other studies related to head and neck cancer indicated that moderate to severe mucositis was one of the strongest predictive characteristics for malnutrition, significant for malnutrition at the 7-week and the 3-month follow-ups. The OR for mucositis was three times higher compared to the no mucositis group [30], this is the first study to prove a similar association in oral cGVHD patients. Patients with mucositis present with weight loss more frequently than patients without mucositis [31]. Severe mucositis can have long-term effects on the nutritional status and standardized follow-up with repeated weight measurements for this sub-group should be strongly considered. It has been reported that early nutritional intervention improves the oral mucositis and nutritional status of patients [32] and can prove highly beneficial for patients that are likely to lose further weight if they were to develop oral cGVHD.

The findings of this study must be seen in light of some limitations. First, the study is retrospective in nature, and it was difficult to assess the length and severity of the symptoms. Chart abstraction was done by a single examiner. Another limitation of our study was selection bias, the selection of exposure cohort, which should have been subjects with oral cGVHD alone. However, due to the rarity of such a cohort, we had to include oral cGVHD with other organ involvements. Hence it is difficult to generalize our results to the population with oral cGVHD alone. Nevertheless, we addressed this concern by differentiating between oral cGVHD with and without GIT involvement, in other words with other organ systems such as skin, and eye involvement. We found that there was no significant difference between the oral and GIT involved cohort and the oral and non-GIT involved cohort in terms of malnutrition indicating that oral cGVHD may be a significant factor contributing to the malnutrition (Table 5). Prospective clinical studies that include newly diagnosed oral cGVHD patients alone, providing longitudinal monitoring of the nutritional status, and assessing early therapeutic intervention are needed. The weight change pattern is difficult to assess since we are evaluating two weights for the patients–current visit weight and pre-SCT weight. It may be argued that we missed a patient at their weight peak. While this is a true concern, we feel that the weight change data are valid when taken as a whole. A large percent weight loss correlates well with a low BMI when compared with the BMI data, which suggests that patients who have lost a considerable amount of weight are malnourished regardless of their starting weight pre-SCT or a potentially missed peak in weight.

Despite these limitations, the study contributes to a better understanding that there is substantial malnutrition in patients who have developed oral cGVHD after HSCT and that oral mucositis is a significant predictor of weight loss above 5%.

## Conclusion

Within the limitations of this study, patients with oral cGVHD had a higher risk of being underweight compared to the non-GVHD and non oral-cGVHD groups. Oral mucositis was an independent predictor of weight loss in patients who developed oral cGVHD. Our findings can facilitate the identification of high-risk patients that are likely to become malnourished, and do not recover healthy weight before developing cGVHD and losing more weight. Weight loss is an indication for initiating oral cGVHD treatment and should be included in the management strategies. Preventive measures early during treatment and in time between the resolution of mucositis and development of cGVHD may have a significant effect on the patient outcome and quality of life. We believe that early recognition and monitoring of oral mucositis and its complications, including mouth pain, ulcers, and infections before developing cGVHD, may improve the overall outcomes.
